# Environmental filling materials based on phosphogypsum powder with municipal solid waste incineration ash

**DOI:** 10.1038/s41598-022-26731-9

**Published:** 2023-01-10

**Authors:** Longlong Yin, Qing Guo, Xiao Wang, Jing Yuan, Qianfeng Zhang

**Affiliations:** 1 Institute of Molecular Engineering and Applied Chemistry, Anhui University of Technology, Ma’anshan, 243002 China; 2 POWERCHINA Hebei Engineering Corporation Limited, No. 107 Tabei Road, Shijiazhuang, China; 3 Department of Civil Engineering, Tongling University, No. 4, Cui Hu Road 1335, Tongling District, 244000 Anhui China; 4 University of Alberta, Edmonton, T6G 1H9 Canada

**Keywords:** Environmental chemistry, Environmental sciences, Chemistry, Engineering

## Abstract

A new building filling materials (NBFM) using phosphogypsum and municipal solid waste incineration (MSWI) fly ash is prepared in this paper. The effects of MSWI fly ash dosage and MSWI fly ash water washing pretreatment on mechanical properties, setting time, metal leaching, hydration products and microstructure of NBFM are analyzed by a range of experimental studies. The results indicate that the mechanical properties, setting time and the density of micro interface of NBFM are optimal when the MSWI fly ash dosage is 3%. The mechanical properties of NBFM rise and the condensation time and leaching concentration of heavy metals decline after washing the MSWI fly ash. With the increase of the curing age, the metal element leaching of NBFM decreases, and when the curing age is 7 days, the solidification effect of NBFM on most metal elements meets the standard of Chinese code (GB5085.3-2007). The feasibility of MSWI fly ash and phosphogypsum as filling materials for building engineering is verified, and the change of macroscopic properties of NBFM is explained as well.

## Introduction

Phosphogypsum is one of the industrial by-products of wet-process phosphoric acid productions, that producing one ton of phosphoric acid can bring 4–5 tons of phosphogypsum. The annual production of phosphogypsum from the phosphorus fertilizer industry worldwide is about 300 million tons^1^. An amount of hoarded phosphogypsum not only occupies land and pollutes the environment, but also the heavy metals in phosphogypsum will flow into the groundwater with rainwater, resulting in pollution to water resources. Thus, the effective utilization of phosphogypsum has received extensive attention^2–5^^.^

The relevant experimental studies^6–8^ have shown that the phosphogypsum was of self-consolidating properties. The use of phosphogypsum for filling materials is feasible and has high value for natural resource conservation, environmental protection, and economic development^9–11^^.^ To improve the application of phosphogypsum filling materials (PFM) in building engineering, some scholars have focused on the physical behavior of the PFM. Gu^12^ conducted an experiment to study the influence of phosphogypsum on PFM. The results revealed that with the increase of phosphogypsum content, the fluidity of PFM increases, and the setting time increases. Mashifana^13^ analyzed the influence of curing method and phosphogypsum content on PFM. The results show that high temperature curing can improve the strength of PFM, and the strength of PFM is the highest when the phosphogypsum content is 30%. Jiang^14^ used phosphogypsum as a binder to prepare PFM. The results indicate that the compressive strength and flexural strength of PFM after 2 h were 3.2 MPa and 1.6 MPa respectively, which can meet the strength standard of the Chinese code. Chen^15^ used phosphogypsum as the base material to prepare PFM. The influence of cement, silica powder and quicklime on PFM strength was analyzed. Results indicated that under the activation of Portland cement, micro-silica powder and quick lime, the strength of PFM increases at the later stage, and strength of PFM was 20 MPa at 28 days.

Municipal solid waste incineration (MSWI) ash is a hazardous waste^16–19^, with the rapid application of waste incineration technology, discharge of MSWI ash in fast growth, but the safety of the landfill of MSWI ash ability is not enough. After the loss of regulation control, much incineration that MSWI ash directly into the environment would contaminate soil and groundwater, bringing huge pollution risk to the environment. MSWI ash including bottom ash and fly ash. The application of bottom ash has great economic and environment benefits. Therefore, Dou^20^ has analyzed the properties, treatment methods and application status of MSWI bottom ash by experiment. The results indicate that MSWI bottom ash as a low strength aggregate has great potential. Davinder^21^ has discussed the effect of cement and fiber on the compaction and strength behavior of MSWI bottom ash. Results show that the maximum dry unit weight of the bottom ash decreases and the optimum moisture content raises due to the addition of cement and fiber. Also, adding fibrin can reduce the hardness of MSWI bottom ash. Jing^22^ investigated the influence of mechanical activation on the characteristics of MSWI bottom ash-cement paste. The results demonstrate that mechanical activation increased the compressive strength of the MSWI bottom ash-cement paste significantly, which increased by 14% when the milling time was 30 min. Laura^23^ used an advanced dry recovery method to separate non-ferrous and ferrous metals from MSWI bottom ash and produce aggregate products with different particle sizes, which is significant for the recycling of MSWI bottom ash. Pravez^24^ uses bottom ash and cement from municipal solid waste incineration in the manufacture of bricks. The results showed that the minimum water absorption and minimum compressive strength criteria of bricks are also satisfied when the cement is substituted for 6% of MSWI bottom ash.

Meanwhile relevant scholars have studied MSWI fly ash^25–28^, and the results show that MSWI fly ash and cement have similar chemical compositions and can be applied as admixtures in gelling systems. But currently, the main cause limiting the resource utilization of MSWI fly ash is that the heavy metals and dioxins in MSWI fly ash are very polluting to the environment^29–31^. Whether the self-consolidating ability of phosphogypsum and the partial hydration activity of MSWI fly ash can be used to encapsulate the heavy metals and dioxins in the colloid to make new filling materials for building engineering. This is the focus of this article and a new method of utilizing phosphogypsum and MSWI fly ash resources for co-processing.

To verify the feasibility of preparing new building filling materials (NBFM) with phosphogypsum and MSWI fly ash. In this paper, a series of experimental studies were carried out on NBFM, and the influence of MSWI fly ash dosage, MSWI fly ash washing pretreatment and other factors on mechanical properties, setting time, heavy metal leaching, hydration products and microscopic appearance of NBFM were analyzed. Furthermore, the relationship between microscopic appearance and macroscopic properties of NBFM was established.

## Experiment

### Experimental materials

**Figure 1:**
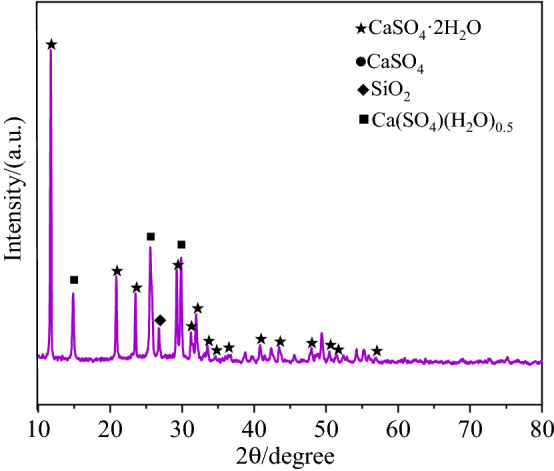
Physical phase composition of phosphogypsum.

The materials used are phosphogypsum, MSWI fly ash, sodium sulfate solution, glass fiber and water. The phosphogypsum is from Guizhou Yitian New Technology Co; The MSWI fly ash from Nanjing Waste Incineration Power Plant. Table 1 presents the heavy metal concentrations of MSWI fly ash and phosphogypsum samples. Table 2 shows the physical indexes of phosphogypsum determined by "Geotechnical Test Regulations"^32^. The chemical composition of phosphogypsum was analyzed by X-ray fluorescence spectrometry, as shown in Table 3. XRD patterns of phosphogypsum are shown in Fig. 1. As can be seen from Fig. 1, phosphogypsum is mainly composed of dihydrite and hemihydrite. The chemical composition of waste incineration MSWI fly ash is shown in Table 4. The length of glass fiber is 1–2 cm, the density is 2.6 g/cm, and the elongation after fracture is 3.4%. The sodium sulfate solution was prepared with analytically pure anhydrous sodium sulfate and tap water.

**Table 1:** Heavy metal content of phosphogypsum and MSWI fly ash.

Phosphogypsum	Contaminants	Cd	Cu	Hz	Pb	As	Zn	–	–
	Content(mg/kg)	0.15	0.48	0.53	29.2	1.32	0.21	–	–
MSWI fly ash	Contaminants	Cu	Zn	Mn	Pb	Cd	Ba	Ni	Cr
	Content(mg/kg)	16.5	48.3	32	84.6	15.6	5.4	36.4	76.3

**Table 2:** Basic physical properties of phosphogypsum.

Porosity (%)	Proportion	Bulk density(g cm^−3^)	Free water content (%)	Crystallization content (%)
50.23	2.34	1.19	21.22	6.23

**Table 3:** Chemical composition of phosphogypsum (%).

CaO	SO_3_	F	P_2_O_5_	Crystal H_2_O	Free H_2_O
29.7	38.1	0.21	0.32	4.96	22.80
Fe_2_O_3_	Al_2_O_3_	MgO	SiO_2_	PH	–
0.03	0.21	0.44	3.12	5.23	–

**Table 4:** Chemical composition of MSWI fly ash(%).

CaO	SiO_2_	Na_2_O	SO_3_	K_2_O
42.8	16.70	7.98	6.7	5.83
MgO	Fe_2_O_3_	Al_2_O_3_	CI	Other
1.75	1.32	1.04	14.6	2.41

Table 2 shows that phosphogypsum has large porosity and high free water content. Therefore, phosphogypsum may be converted to hemihydrous gypsum during replacement.

Table 3 indicates that phosphogypsum is mainly composed of C, S, O and other oxides, among which the content of active ingredients such as CaO and SiO_2_ is as high as 33%. Refer to Chinese specification (GB/T 9776-2008)^33^, phosphogypsum has the property of self-cementing curing.

Based on the Chinese norms (HJ/1134-2020)^34^ and Table 4. MSWI fly ash is the product of CaO-SiO_2_-Al_2_O_3_ system formed at high temperature, which has a certain pozzolash effect and can be added into phosphogypsum as admixture.

## Experimental procedures

Experimental procedures are mainly followed three steps: MSWI fly ash washing treatment, Mixing design and production process. The detailed procedures of these three steps are listed as below.

### MSWI fly ash washing treatment

The MSWI fly ash was pretreated by water washing. To compare the effects of washed and non-washed MSWI fly ash on mechanical properties, setting time, heavy metal leaching amount and microstructure of NBFM. The main procedures are as follows: first, MSWI fly ash and pure water are mixed at a solid-liquid ratio of 1:8^35,36^, and then placed on the TCLP rotating oscillator to oscillate at a frequency of 30 r/min for 30 min^37,38^. After the oscillation, it stands for 12 h. Finally, the surface moisture was removed, and the bottom MSWI fly ash was placed in a 105 ℃-drying box for 24 h to obtain the MSWI fly ash particles.

### Mixing design

Some experiments and theoretical studies are used^39–41^. The mixing ratio of NBFM is determined: the ratio of water to binder material is 0.39, in which the binder material is phosphogypsum and MSWI fly ash, the dosage of glass fiber and sodium sulfate solution was 0.3% and 2.5% of the binder material respectively. The mixing design of binder material is shown in Table 5.

**Table 5:** Mixing design of binder material.

Specimens ID	Phosphogypsum dosage (%)	MSWI fly ash dosage (%)	MSWI fly ash washing/no- washing
S_1_	100	0	No-washing
S_2_	97	3	No-washing
S_3_	95	5	No-washing
S_4_	90	10	No-washing
S_5_	70	30	No-washing
S_6_	97	3	Washing

### Production method

**Figure 2:**
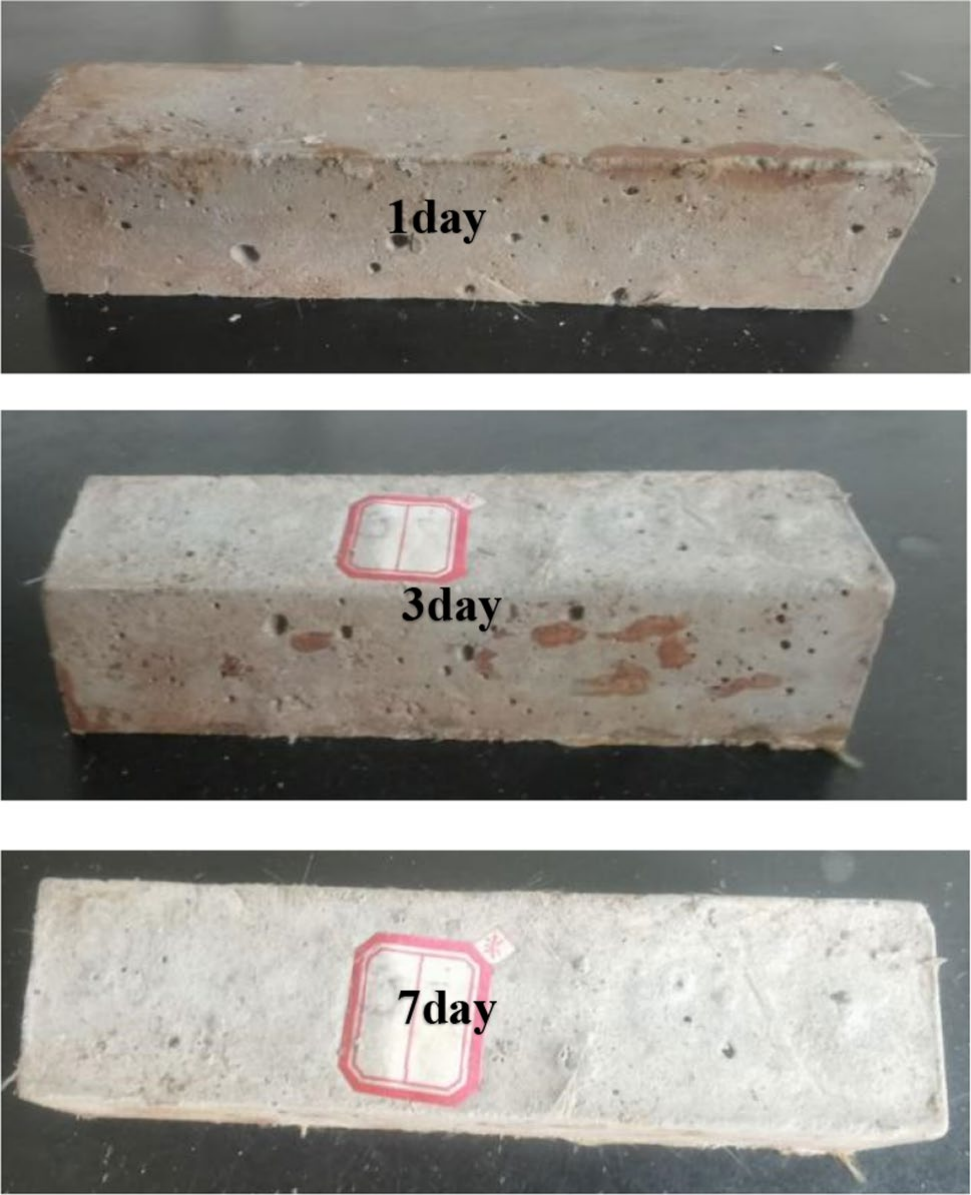
NBFM specimens of 1d, 3d, 7d maintenance age.

First, weigh the material according to the mixing design, and pour the material into the agitator and stir it with water for 30–50 s to get a uniform filling slurry. The prepared paddle slurry was then put into the standard triple mold of 40 mm × 40 mm × 160 mm and allowed to sink naturally. After initial coagulation of the slurry, the surface of the slurry was scraped flat and cured at room temperature for 24 h. Finally, the molded NBFM was demold and transferred to the curing room for 1 day, 3 day and 7 day. The forming diagram of NBFM at different maintenance ages are shown in Fig. 2.

## Experimental methods

### Mechanical properties

Mechanical properties measured from the coupon tests according to Chinese code "Determination of mechanical properties of building gypsum"^42^ (GB/T17669.3-1999). The instrument used for the test was a cement pressure tester DP-300C. The size of the specimen is in 40 × 40 × 160 mm prismatic shape, the support spacing of the test fixture is 100 mm, and the loading speed is 0.03–0.06 MPa/s. The flexural strength of the specimen is calculated by Eq. (1)^42^. The block with finished flexural strength is tested for compressive strength. The fractured specimen was put into a square clamp of 40 × 40 mm and tested according to the loading speed mentioned above. The compressive strength of the specimen is calculated by Eq. (2)^42^.

1 $$f_{{{\text{cf}}}} = \frac{6M}{{b^{3} }} = 0.00234F$$

where, $f_{{{\text{cf}}}}$ is the flexural strength (MPa) of the specimen, $F$ is the fracture load (N) of the specimen, $M$ is the flexural moment ($N \cdot {\text{m}}$) of the specimen when it breaks, and $b$ is the side length (mm) of the square section of the specimen.

2 $$f_{{{\text{cu}}}} = \frac{F}{S}$$

where, $f_{{{\text{cu}}}}$ is the compressive strength (MPa) of the specimen and $S$ is the bearing area (mm^2^) of the specimen.

### Setting time

The setting time has been done according to GB/T17669.4-1999 "Determination of physical Properties of building gypsum/Net Slurry"^43^. The specific test process is as follows: first, uniform filling slurry is obtained in accordance with the production process. Then the slurry is poured into the ring die device, and the bottom plate of the device is raised so that the slurry is flush with the upper end of the ring die. Place the ring mold filled with slurry under the steel needle of the consistency meter, make the tip contact with the surface of slurry, and then quickly loosen the fixing screw on the rod, so that the needle can freely insert into slurry. From the beginning of the material contact with water, the time that the steel needle cannot touch the bottom plate for the first time, that is, the initial setting time of the specimen. The final setting time of the specimen is the time experienced from the contact between the material and water to the first time when the steel needle is inserted into the slurry depth of no more than 1mm.

### Heavy metal leaching

The leaching of heavy metals was carried out according to the sulfuric acid method in the Leaching Toxicity Identification of Hazardous Waste Identification Standard (EPA SW-846 Test Method 1311^44^, GB/T5085.3-2007)^45^. The specific testing process is as follows: to begin with, the sample is broken and finely ground to 5 mm. Then, concentrated sulfuric acid and concentrated nitric acid with a mass ratio of 2:1 was mixed into water to prepare an extract with a pH of about 3.2. Then the sample and the extraction agent were mixed in the extraction bottle with the ratio of liquid to solid of 10 L:1 kg. After that, the extraction bottle was tightly capped and placed in a horizontal oscillation device for 18 h at a speed of 30 r/min and a tilt angle of 23°. Finally, the leaching solution was collected and put into the inductively coupled plasma mass spectrometer (ICM-MS, Agilent 7500CX) to determine the dosage of heavy metals.

### XRD

XRD instrument (D8ADVANCE) and powder samples were used in the test. The scanning angle was set at 5°–90° and the scanning speed was set at 10°/min. The specific production process of the sample is as follows: to begin with, the material is made and molded according to the steps and cured to the 7 days. Then, square samples with a radius of 0.5 cm were cut with a cutting machine and cleaned with anhydrous alcohol. After drying, the samples were ground to powder with agate mortar. Finally, put the powder sample into the instrument for analysis.

### SEM

SEM instrument (JSM-6490LV) was used in this experiment. The observation multiple was 1000 times. The specific production process of the sample is as follows: first of all, the material is made and molded according to the steps and cured to the 7 days. A sample no more than 15 mm in diameter and no more than 5 mm in thickness was then removed with a cutting machine and cleaned with anhydrous alcohol. After that, the sample was placed in a drying box at 95 ℃ and vacuum dried to interrupt the hydration reaction. Before observation, the sample shall be plated with gold, carbon, platinum, and other coating materials with thickness of about 10–30 nm. After coating, it can be placed on the sample table for observation and analysis.

## Results and discussion

**Figure 3:**
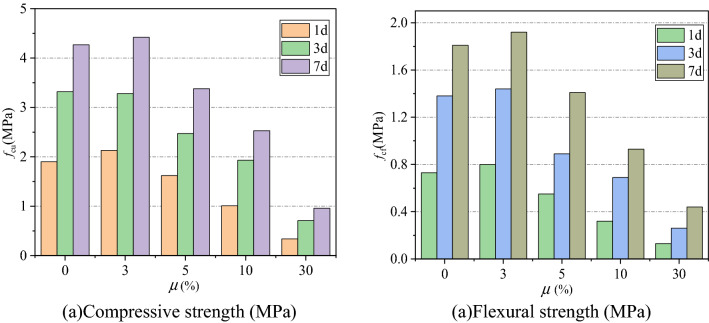
Influence of MSWI fly ash dosage on mechanical properties of NBFM.

**Figure 4:**
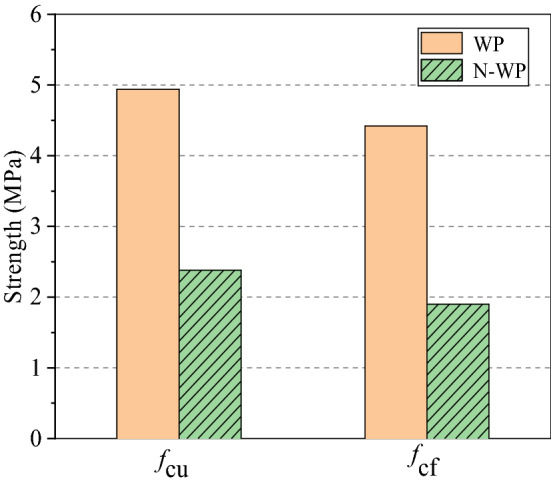
Influence of MSWI fly ash washing pretreatment on mechanical properties of NBFM.

**Figure 5:**
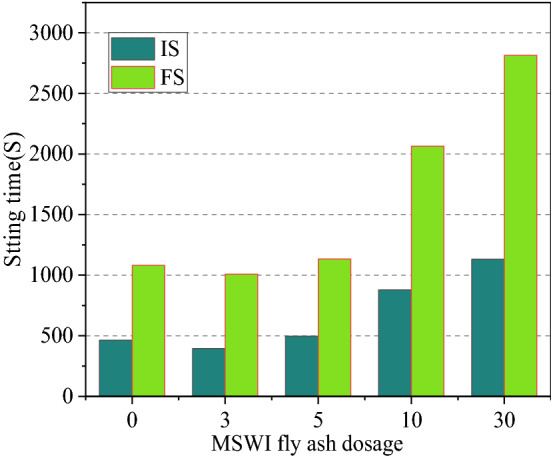
Influence of MSWI fly ash dosage on setting time of NBFM.

**Figure 6:**
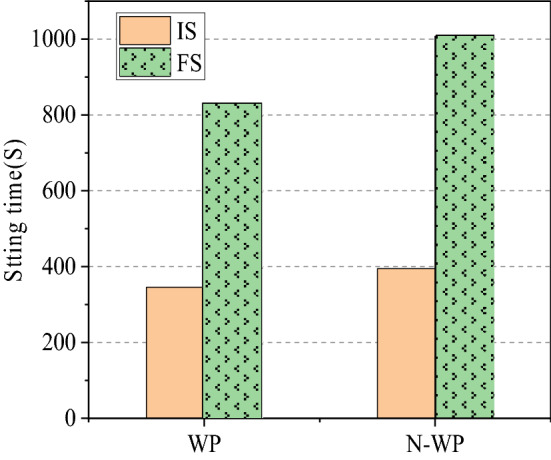
Influence of water washing pretreatment on condensation time of NBFM.

The mechanical properties and setting time of NBFM are the basic performance indexes that affect building construction. The effects of MSWI fly ash dosage and water washing pretreatment on the mechanical properties and setting time of NBFM are discussed respectively. In the Fig. 3, $\mu$ stands for MSWI fly ash dosage of NBFM. $1{\text{d}}$,$3{\text{d}}$,$7{\text{d}}$ represent the conservation age of 1 day, 3 days and 7 days respectively. $f_{{{\text{cu}}}}$ and $f_{{{\text{cf}}}}$ represent the compressive strength and flexural strength of NBFM respectively; ${\text{WP}}$ in the Fig. 4 stands for MSWI fly ash washing, while ${\text{N}} - {\text{WP}}$ stands for MSWI fly ash not washing. ${\text{IS}}$ represents initial setting time and ${\text{FS}}$ represents final setting time in the Figs. 5 and 6.

### Mechanical property

#### Dosage of MSWI fly ash

The influence of MSWI fly ash dosage on mechanical properties of NBFM is shown in Fig. 3. The compressive strength and flexural strength of the NBFM increase first and then decrease as the increase of MSWI fly ash dosage. When the MSWI fly ash dosage is 3%, the NBFM has the best mechanical properties, and the compressive strength and flexural strength are 4.42 MPa and 1.9 MPa, respectively. This is mainly because when the MSWI fly ash dosage is 3%, the fine particles in the MSWI fly ash can provide the crystallization nucleation point for the hydration products in the NBFM, promoting the generation of hydration products, resulting in the compressive strength and flexural strength of the NBFM appear an increase trend. However, with the increasing of MSWI fly ash dosage, loose and porous dust particles in the NBFM are increasing, which reduces the water consumption required for phosphogypsum based reaction and inhibits the formation of phosphogypsum based crystals^46^. In addition, with the increase of MSWI fly ash dosage, the NBFM contains more and more organic components such as chloride and sulfide, leading to the gradual destruction of the structure of hydration products in the NBFM^47^. Therefore, the strength of the NBFM is significantly reduced.

#### MSWI fly ash washing pretreatment

Figure 4 shows the influence of MSWI fly ash washing pretreatment on mechanical properties of NBFM. Compared with non-washed MSWI fly ash, the MSWI fly ash washed with water can significantly improve the mechanical properties of the NBFM. This is mainly because of water-soluble components in MSWI fly ash composition such as Na, K, Cl, Ca will deposit on the surface of MSWI fly ash particles, easy to be removed after sufficient water washing, and Si and Al components in MSWI fly ash particles at the center of the matrix, with fewer particles peripheral components, makes the core components of the exposed surface increases, lead to increase the block the activity of the binder composition, degree of hydration enhance^48^. Therefore, the mechanical properties of the NBFM increase.

### Time of coagulation

#### Dosage of MSWI fly ash

The influence of MSWI fly ash dosage on the setting time of NBFM is shown in Fig. 5. With the increase of MSWI fly ash dosage, the initial setting time and final setting time of the NBFM show a development trend of first decreasing and then increasing. This is mainly because the small radius of Cl− ions in MSWI fly ash can penetrate the encapsulation of hydration products, leading to the reverse diffusion of OH− ions in NBFM and accelerating Ca(OH)_2_ precipitation. Therefore, the hydration reaction in the early stage of the NBFM is enhanced and the condensation time is shortened. However, with the increasing of MSWI fly ash dosage, Zn, Pb, Cu, Cr and other heavy metal elements in the NBFM keep increasing, and the heavy metal will inhibit the condensation and hardening of the NBFM^48^, leading to the gradual increase of the condensation time of the NBFM.

#### MSWI fly ash washing pretreatment

Figure 6 shows the influence of washing pretreatment of MSWI fly ash on the condensation time of the NBFM. The initial and final coagulation time of the NBFM after washing pretreatment of MSWI fly ash are reduced. The main reason is that the content of active components such as CaO, Al_2_O_3_, SiO_2_ in the MSWI fly ash increases after washing^49^, which leads to a faster process of hydration reaction in the NBFM. Therefore, the initial and final coagulation time of the NBFM decreased significantly with the washing pretreatment.

In conclusion, when the MSWI fly ash dosage is 3%, the strength and setting time of the NBFM meet the standards of grade 2.0 building gypsum in Chinese code (GB/T9776-2008)^50^. The basic physical properties of the NBFM are significantly enhanced after washing the MSWI fly ash with water. Therefore, it is feasible to apply the NBFM to building engineering with higher quality after water washing pretreatment.

### Heavy metal leaching concentration

This paper has demonstrated that the basic physical properties of the NBFM meet the standard for use as filling materials. However, whether phosphogypsum can solidify heavy metals in MSWI fly ash needs to be analyzed and evaluated for its heavy metal leaching characteristics. Therefore, based on mechanical property experiment and time of coagulation experiment, this research conducted heavy metal leaching experiments on S_2_ and S_6_ samples and analyzed the influence of curing age and MSWI fly ash washing pretreatment on metal leaching concentration of NBFM.

### Curing age

**Figure 7:**
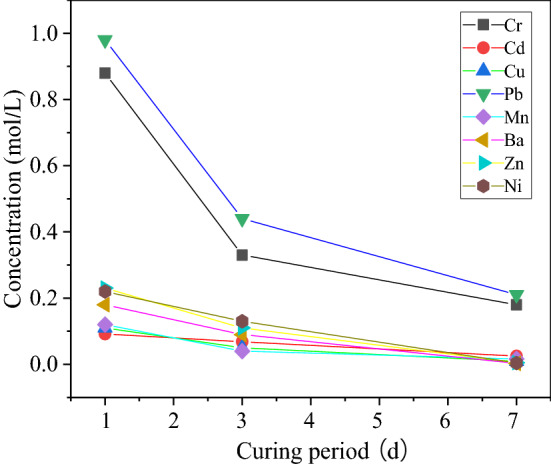
Influence of curing age on leaching of heavy metal from NBFM.

The change of heavy metal leaching amount in the NBFM during the curing period (day) is shown in Fig. 7. The change pattern of the curing age in the Fig. 7 is S_3._ With the increase of curing age, the leaching number of heavy metals in the NBFM gradually decreases. When the curing age is 7 days, the leaching concentration of most heavy metals in the NBFM is relatively low, and only the concentration of Cr and Pb is higher than the standard value of Chinese code (GB5085.3-2007)^45^. This is mainly because, with the increase of curing age, the heavy metals in MSWI fly ash react with the hydration products in the NBFM by adsorption, ion exchange, chemical reaction, surface complexation and other ways, and constantly form hydroxides and complexes, remaining on the crystal surface. Therefore, the amount of metal leaching in the NBFM decreases continuously. Phosphogypsum has a good curing effect on most metal elements in MSWI fly ash and it has a certain solidifying effect on heavy metals in waste MSWI fly ash. This method could be a new way to treat phosphogypsum and MSWI fly ash from MSW incineration. However, concentration of Cr and Pb metals is relatively high, although the leaching concentration has decreased, it still does not meet the requirements of the code, and further research is needed to reduce the concentration of Cr and Pb.

### MSWI fly ash washing pretreatment

**Figure 8:**
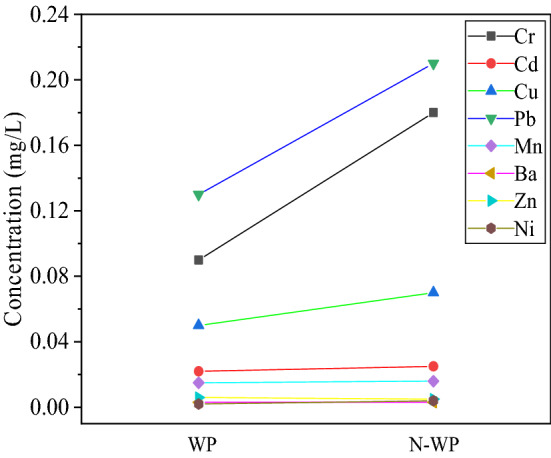
Influence of MSWI fly ash washing pretreatment on heavy metal leaching of NBFM.

Figure 8. shows the influence of washing pretreatment on the leaching amount of heavy metal in the NBFM. Water washing can effectively reduce the concentration of most metal elements in the NBFM. This is mainly because, in the process of washing, some water-soluble heavy metals such as Cu, Zn, As and Hg in the MSWI fly ash gradually dissolve under the action of vibration, so the concentration of heavy metals after washing gradually decreases. As can be seen from the above, phosphogypsum has a good solidification effect on the washed MSWI fly ash, and the use of washed MSWI fly ash as an auxiliary material for phosphogypsum is safe.

In conclusion, phosphogypsum has a certain curing effect on heavy metals in waste MSWI fly ash, but the leaching concentration of some metal ions (such as Cr, Pb, etc.) is still higher than the standard requirements. Therefore, the leaching concentration of heavy metals in MSWI fly ash should be further reduced by optimizing the curing process, adding additives, or pickling MSWI fly ash before curing^51–54^^.^

### Micro-mechanical discussion

To establish the relationship between the macroscopic properties and microscopic appearance of the NBFM. SEM and XRD techniques were used to reveal the change mechanism of basic physical properties of NBFM from the two aspects of microstructure and hydration products.

### XRD analysis

Compared with standard cards, Gypsum (CaSO_4_·2H_2_O), Anhydrite(CaSO_4_), Quartz(SiO_2_) and hydrated lime (Ca(OH)_2_) were mainly detected in the diffraction patterns of NBFM, while the hydration products calcium silicate hydrate (C–S–H) were precipitated in the form of gel. Therefore, no characteristic peak of C–S–H was detected.

### Dosage of MSWI fly ash

**Figure 9:**
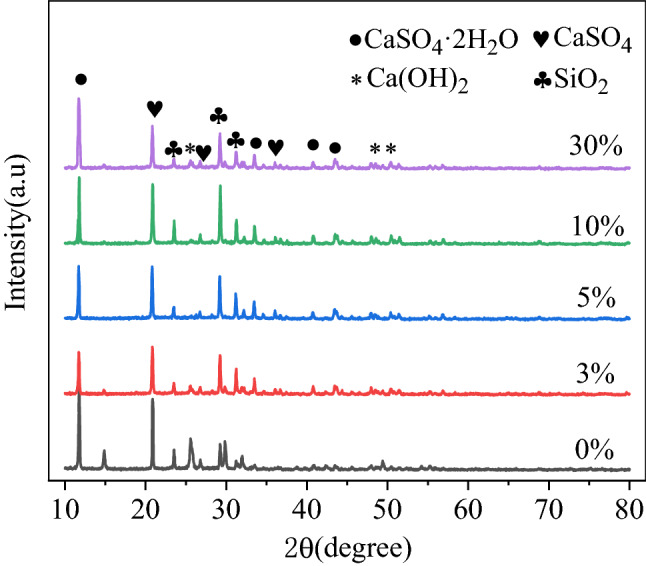
Influence of MSWI fly ash dosage on crystal dosage in NBFM.

The influence of MSWI fly ash dosage on crystal dosage in the NBFM is shown in Fig. 9. With the increase of MSWI fly ash dosage, the peak strengths of CaSO_4_·2H_2_O, CaSO_4_ and SiO_2_ in the NBFM decrease first and then increase. This indicates that the hydration reaction of the NBFM is the largest when the MSWI fly ash dosage is 3%. This is mainly because MSWI fly ash has a certain pozzolanic reaction. As the MSWI fly ash dosage increases, the active components in MSWI fly ash can accelerate the hydration reaction of the NBFM. But as the dosage of MSWI fly ash continues to increase, the dosages of heavy metal ions and sodium chloride and other salt compounds in the NBFM gradually increase, leading to the reaction between hydration products and metal ions and salt compounds. For example, when Cr in MSWI fly ash exceeds 1.56%, part of CaSO_4_ will decompose and generate CaCrO_4_, leading to a trend of gradually decreasing hydration reaction of the NBFM^55^. Therefore, the strength of the NBFM increases and then decreases with the increase of MSWI fly ash dosage.

### Curing age

**Figure 10:**
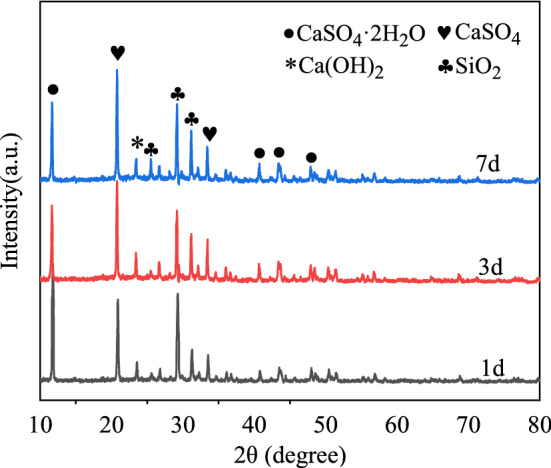
Influence of MSWI fly ash dosage on crystal dosage in NBFM.

Figure 10 shows the influence of curing age on the crystal dosage of the NBFM. With the increase of curing age, the peak strength of CaSO_4_·2H_2_O, SiO_2_ and Ca(OH)_2_ decreases gradually, and the decrease is relatively when the curing age is between 1 day and 3 days. This indicates that the hydration reaction of the NBFM mainly occurred in the early stage. This is because the MSWI fly ash contains nondeterminacy SiO_2_^16^. A proper amount of SiO_2_ can induce the hydration reaction between CaSO_4_·2H_2_O and CaO to generate CaSO_4_ and a small amount of Ca(OH)_2_, while Ca(OH)_2_ and SiO_2_ have volcanic ash effect to generate C–S–H gel, resulting in CaSO_4_·2H_2_O in NBFM. The peak intensities of SiO_2_ and Ca(OH)_2_ decrease gradually. Meanwhile, with the increase of curing age, hydration products gradually precipitate on the surface of gypsum particles and MSWI fly ash particles, hindering the hydration reaction of the NBFM^56,57^. Therefore, with the increase of curing age, the hydration reaction of the NBFM gradually weakens.

### MSWI fly ash washing pretreatment

**Figure 11:**
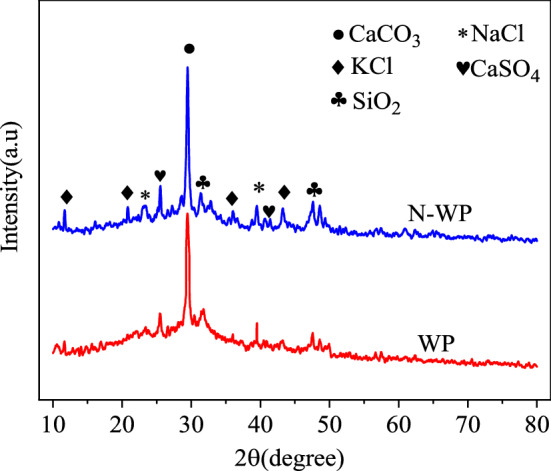
Influence of water washing pretreatment on crystal dosage of MSWI fly ash.

Figure 11 shows the influence of water washing pretreatment on the crystal dosage of MSWI fly ash. NaCl and KCl in the MSWI fly ash have been basically removed after washing, and the physical phases of the MSWI fly ash after washing mainly exist in the form of CaCO_3_, SiO_2_, and CaSO_4_, and the miscellaneous peaks of the MSWI fly ash after washing are reduced. This is mainly because washing removes many compatible substances in MSWI fly ash, such as potassium salts and chlorine salts. The crystalline phase of heavy metals is not detected in the XRD pattern, which may be because the metal structure is small and usually wrapped by other mineral components^58^, so the characteristic peak of the metal phase is not detected.

### SEM analysis

The microstructure of the NBFM has a decisive effect on its basic physical properties. Therefore, the influence law of MSWI fly ash dosage, curing age and washing pretreatment on the microscopic appearance of the NBFM are analyzed. The micromorphology of the NBFM mainly includes gypsum crystal, SiO_2_ crystal, C–S–H gel, Ca(OH)_2_ crystal and some gypsum and MSWI fly ash particles.

### Dosage of MSWI fly ash

**Figure 12:**
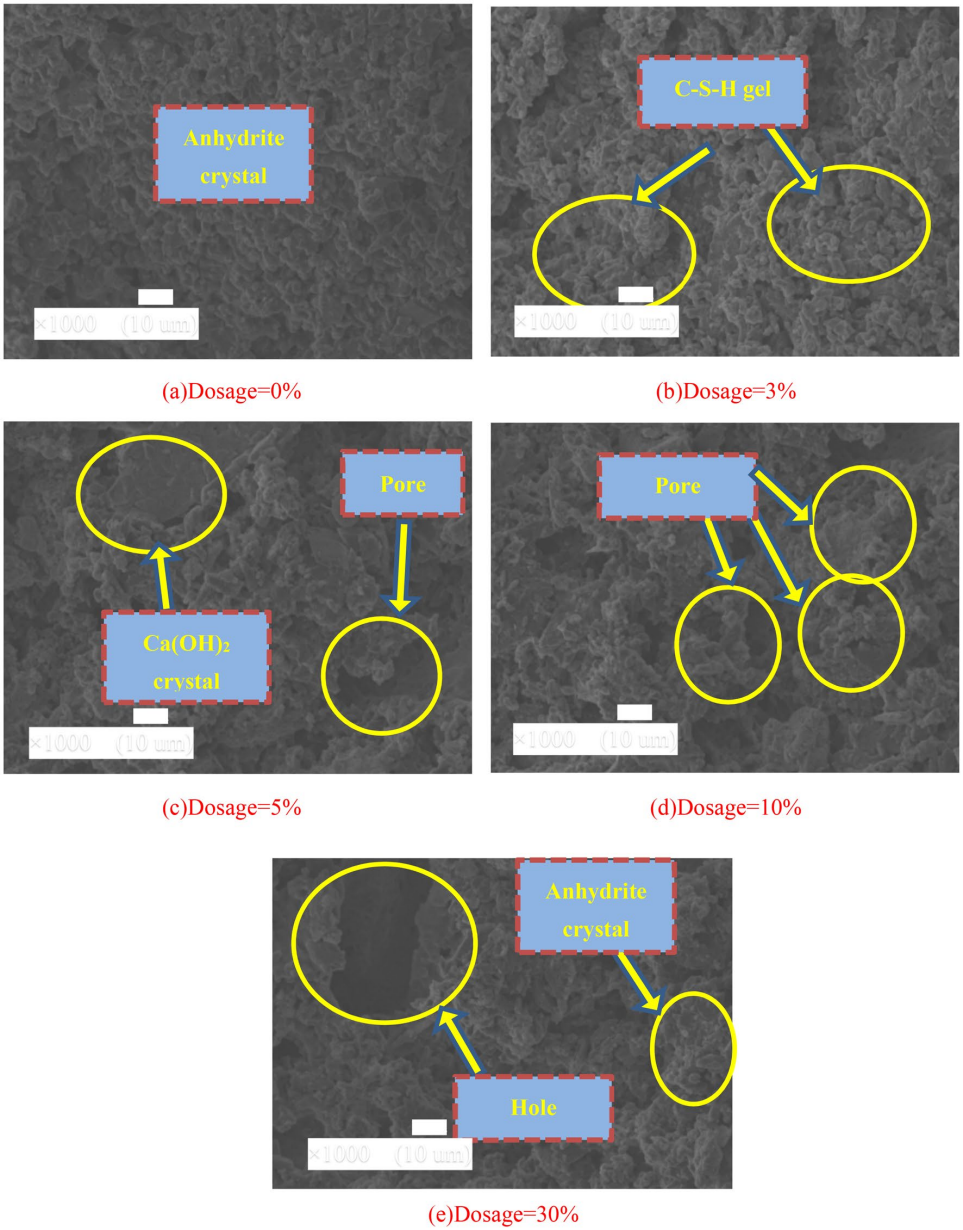
Influence of MSWI fly ash dosage on micro-features of NBFM.

Figure 12 shows the influence of MSWI fly ash dosage on the micro-features of the NBFM. When no MSWI fly ash is mixed, there are many evenly arranged anhydrite crystals at the interface of the NBFM, and the interface is dense, and the number of cracks and holes is small. With the increase of MSWI fly ash dosage, interface hydration products gradually increase, and their thickness and compactness increase. When the MSWI fly ash dosage is 3%, the dosage of granular C–S–H gel and flake anhydrite crystal increases in the interface appearance, hydration products uniformly fill the pores at the interface, and the interface compactness increases. With the further increase of MSWI fly ash dosage, the number of interface pores and MSWI fly ash particles increase, and the compactness decreases. When the MSWI fly ash dosage is 30%, holes appear on the interface of the NBFM. This is mainly because MSWI fly ash is composed of tiny particles. With the increase of MSWI fly ash dosage, small particles in MSWI fly ash can induce the process of hydration reaction, accelerate the reaction of calcium sulfate dihydrate with SiO_2_, Ca(OH)_2_, and Al_2_O_3_, resulting in the increase of hydration products at the interface. However, with the further increase of MSWI fly ash dosage, the dosage of dust particles in MSWI fly ash increases significantly, and a large amount of free water is absorbed to the dust surface during the mixing process, resulting in the reduction of free water involved in the hydration reaction. Secondly, SiO_2_ is composed of tiny particles. In the hydration process, SiO_2_ tends to move closer to MSWI fly ash particles along with water molecules^59^, which significantly reduces the activity of the cementitious material. Therefore, MSWI fly ash and phosphogypsum particles at the interface of the NBFM gradually increase. According to the compactness and stability of the microscopic appearance of Fig. 12, the bond strength of the interface of the NBFM is 0% < 3% > 5% > 10% > 30%, which is consistent with the change rule of mechanical properties in mechanical property.

### Curing age

**Figure 13:**
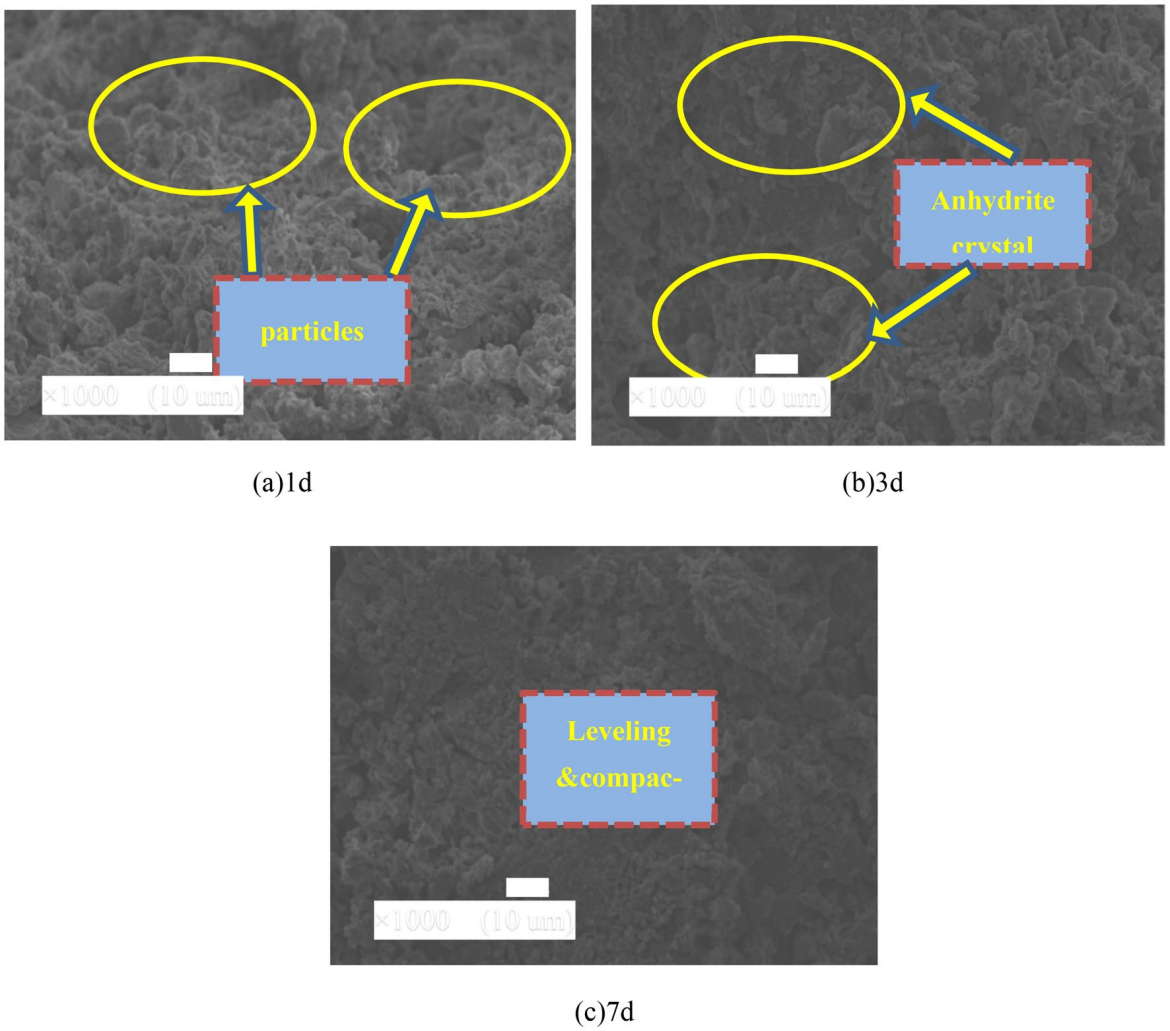
Influence of curing age on micro-features of NBFM.

Figure 13 shows the influence of curing age on micro-features of NBFM. When the curing age is 1 day, hydration products at the interface of the NBFM are mainly anhydrite crystals, C–S–H gel and gypsum particles, which interlock with each other to form a loose skeleton structure, resulting in low density of the interface. With the increase of curing age, the number of C–S–H gel at the interface of NBFM increases, and the interface smoothness increases. When the curing age is 7 days, the interface of the NBFM is relatively smooth, and the number of pores and gypsum particles is small. This is mainly because, with the increase of curing age, calcium sulfate dihydrate reacts with CaO and Al_2_O_3_ in MSWI fly ash to generate granular and fibrous C–S–H gel. The hydration products are interlaced and connected to form a tightly bonded dense entity, leading to the increasing flatness of the interface of the NBFM. Therefore, with the increase of curing age, the mechanical properties of the block gradually increase.

### MSWI fly ash washing pretreatment

**Figure 14:**
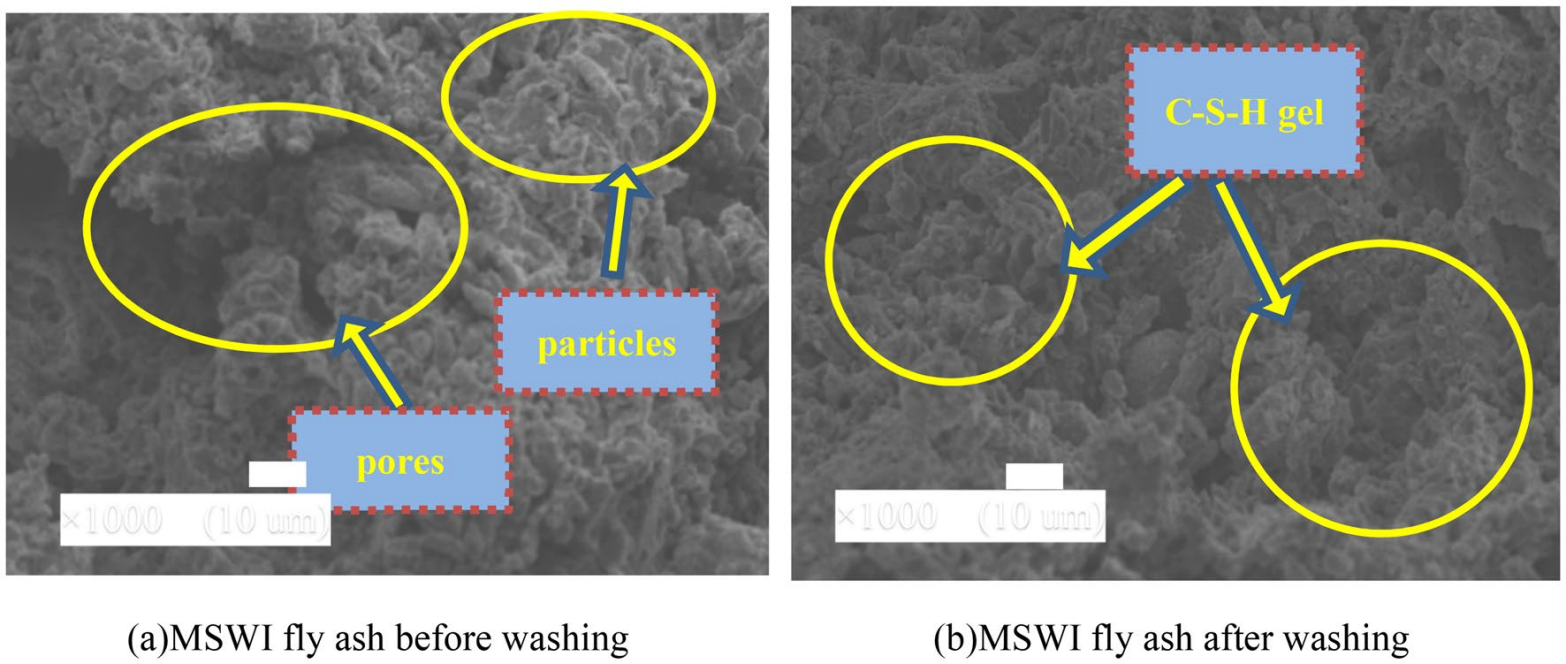
Influence of water washing treatment on micro-features of NBFM.

Figure 14 shows the influence of MSWI fly ash washing pretreatment on the micro-features of the NBFM. The interface of the NBFM before MSWI fly ash washing pretreatment is uneven with holes, cracks, and gypsum particles. After the MSWI fly ash was washed, the hydration products at the interface of the NBFM increased, the interface was compact and uniform, and the number of pores decreased. This is mainly because the soluble substances on the surface of MSWI fly ash particles are removed after water washing, which increases the contact area between active components such as Si and Al in MSWI fly ash and CaSO_4_·2H_2_O, and improves the hydration reaction of NBFM. On the other hand, after water washing, the weight of MSWI fly ash decreases, which leads to the increase of free water content involved in the hydration reaction, and appropriate free water can further promote the hydration reaction of mineral particles in the NBFM^60^. As a result, the contents of Ca(OH)_2_, C-S-H gel and CaCO_3_ in the interface of the NBFM increased significantly, and the compactness increased. According to the compactness and stability of the microscopic appearance of Figure 14, the strength of the interface of the NBFM is (a) < (b), which is consistent with the change rule of mechanical properties in mechanical property.

The connection between macroscopic properties and microscopic features of NBFM was established using SEM and XRD experiments, and the variation of macroscopic properties of NBFM was explained from a microscopic perspective.

## Conclusions

In this paper, MSWI fly ash and phosphogypsum are used to prepare NBFM. By series of experiments, the influence of MSWI fly ash dosage, MSWI fly ash washing pretreatment and other factors on mechanical properties, condensation time, hydration products and microscopic appearance of NBFM were analyzed, and the following conclusions were drawn:

1. With the increase of MSWI fly ash dosage, the mechanical properties of the NBFM show a trend of first increasing and then decreasing, while the initial and final coagulation time of the NBFM show a trend of first decreasing and then increasing. When the MSWI fly ash dosage is 3%, the mechanical properties and setting time of NBFM are the best.
2. After washing the MSWI fly ash, the mechanical properties of the NBFM increased significantly, the initial and final coagulation decreased significantly, and the number of pores in the microscopic boundary of the NBFM decreased, and the flatness increased.
3. When the curing age is 7 days, phosphogypsum has a good curing effect on most metal elements in MSWI fly ash. The concentration of heavy metals in the samples can be significantly reduced by washing with MSWI fly ash.
4. With the increase of curing age, the number of C–S–H gel in the microscopic features of the NBFM increases, and the flatness increases. The appropriate amount of MSWI fly ash can promote the hydration reaction of the NBFM and enhance the density of its micro interface.

In conclusion, this paper proves that the NBFM can be applied to practical engineering through mechanical properties and setting time tests. The effect of phosphogypsum on solidification of heavy metals in MSWI fly ash was verified by heavy metal leaching experiments. The relationship between macroscopic properties and microscopic appearance of NBFM is established by microscopic experiments (Supplementary Information).

## Supplementary Information

Supplementary Information.

## Data Availability

The data used and/or analysed during the current study are available in the supplemental materials or from the corresponding author on reasonable request.
